# Lumbar Disc Herniation: What's New in Surgical Treatment

**DOI:** 10.1055/s-0046-1824521

**Published:** 2026-10-06

**Authors:** Fábio Antônio Vieira, Breno Augusto Giese Ribeiro, Renato Hiroshi Salvioni Ueta, Eduardo Barros Puertas

**Affiliations:** 1 Departament of Orthopedics and Traumatology, Escola Paulista de Medicina, Universidade Federal de São Paulo, São Paulo, SP, Brazil

**Keywords:** diskectomy, herniated disc, sciatica, ciática, discectomia, hérnia de disco

## Abstract

Lumbar disc herniation is one of the main causes of root pain and disability in the adult population, with a significant impact on the healthcare system and labor productivity. Initial management is predominantly conservative, but a portion of patients may evolve with refractory symptoms or progressive neurological deficit, requiring surgical intervention. Over the past few decades, minimally invasive techniques, such as endoscopic surgery, have revolutionized treatment with less tissue damage, accelerated recovery, and lower complication rates. This article reviews the evolution of surgical techniques, highlighting comparative evidence among them and the emerging role of new technologies.

## Resumo

A hérnia de disco lombar é uma das principais causas de dor radicular e incapacidade na população adulta, com impacto expressivo no sistema de saúde e na produtividade laboral. O manejo inicial é predominantemente conservador, mas uma parcela dos pacientes pode evoluir com sintomas refratários ou déficit neurológico progressivo, necessitando de intervenção cirúrgica. Ao longo das últimas décadas, técnicas minimamente invasivas, como a cirurgia endoscópica, revolucionaram o tratamento com menor agressão tecidual, recuperação acelerada e menor taxas de complicações. Este artigo revisa a evolução das técnicas cirúrgicas, destacando evidências comparativas entre elas, bem como o papel emergente de novas tecnologias.

## Introduction

Lumbar disc herniation has a high incidence in the population, being the main cause of low back pain and radiculopathy among adults aged 30 to 50 years. The pathophysiology of hernia is complex and involves progressive biomechanical, biochemical, and structural changes. 1 The degenerative process of the intervertebral disc (IVD) leads to dehydration of the nucleus pulposus (NP), decreased IVD height, and fibrous annulus (FA) overload, which can rupture its fibers. 2 3 The fissures allow the extrusion of the NP content into the vertebral canal, which can cause mechanical compression of the nerve root. 3 Inflammatory mediators released during disc degeneration play a fundamental role, as they can generate root pattern pain even in cases without exuberant compression. 4

Clinically, the patient presents with radiating low back pain along a specific dermatome, which may progress to sensory or motor deficits. 3 5 In cases of bulky hernias or disc sequestrations, the condition may progress to cauda equina syndrome, constituting an emergency due to the risk of permanent sequelae. 6 Levels L4 to L5 and L5 to S1 are responsible for about 90% of lumbar disc hernias, a pattern associated with greater mobility and biomechanical stress in these segments. 2

The natural history of lumbar disc herniation is generally favorable. About 80% of patients show spontaneous improvement within 3 months, and up to 90% in 1 year. 3 Spontaneous resorption of the fragment can occur, especially in migrated or sequestered extrusions, in which the immunological effect on the exposed material accelerates absorption. 7 Conservative treatment is multimodal, consisting of analgesics, anti-inflammatory drugs, physiotherapy, and physical activity, and should be the standard initial approach. 8 9 However, approximately 20% of patients evolve with persistent symptoms after 12 weeks of treatment or present with progressive neurological deficit, making surgical intervention necessary. 3 10 11

Historically, open discectomy has been the classic approach, with high rates of clinical improvement and satisfactory results. 12 Microdiscectomy represented a major technical advance by introducing the microscope to reduce muscle aggression and improve surgical visualization. 13 From the 2000s, minimally invasive techniques, including tubular microdiscectomy and endoscopic discectomy ( Fig. 1 ), were consolidated as effective alternatives, leading to less tissue damage, less bleeding, and faster recovery. 14 15 16

**Fig. 1:**
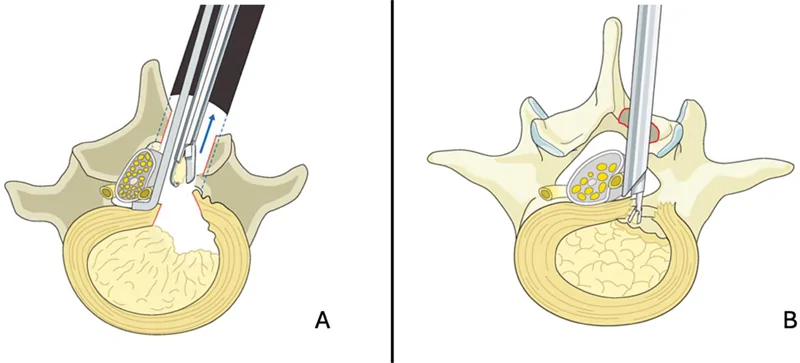
Representation of center-lateral hernia discectomy. ( **A** ) Tubular microdiscectomy. ( **B** ) Endoscopic discectomy. **Source** : Balsano et al. Available from https://surgeryreference.aofoundation.org/spine/degenerative/lumbar/disc-herniation-central-posterolateral/.

Other advances include the development of annular closure devices aimed at preventing relapse in patients with large annular defects, 17 18 and the growing interest in regenerative medicine therapies that explore the potential of signaling cells to restore nucleus pulposus biology and modulate inflammation. 19 In cases of disc hernias with segmental instability or advanced IVD degeneration, arthrodesis continues to play an essential role. 20

Thus, the current scenario of surgical treatment of lumbar hernia is marked by rapid technological development and the convergence of mechanical and biological approaches, expanding the therapeutic arsenal and enabling increasingly individualized strategies.

## Discectomy

### Tubular microdiscectomy

Tubular microdiscectomy employs sequential dilators for atraumatic dilation of the paravertebral musculature. After positioning the working cannula and obtaining microscopic visualization, minimal laminotomy, flavectomy, and removal of the herniated fragment are performed ( Fig. 2 ). 21

**Fig. 2:**
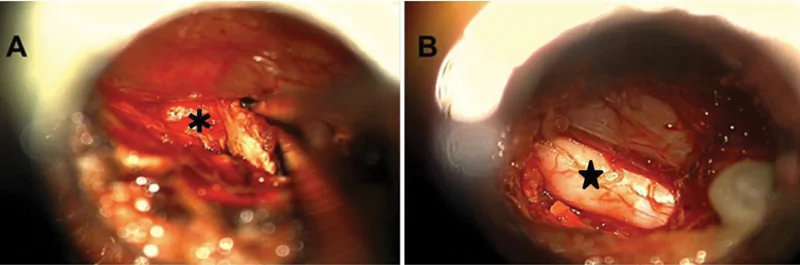
Intraoperative images of tubular microdiscectomy. ( **A** ) Identification of the herniated fragment. ( **B** ) Free nerve after discectomy. ★Lumbar root. ✱Herniated disc.

The main limitations of the tubular technique include a more restricted surgical field, which makes it difficult to expand the exposure if necessary, and lateral hernias or multiple levels, in cases of migrated fragments. 22 The presence of the tubular retractor can make it difficult to manipulate and explore the levels, especially for surgeons unfamiliar with the procedure. Thus, the tubular technique has a longer learning curve than open discectomy. 22

Despite reduced tissue aggression, the clinical results are similar to those of traditional microdiscectomy, with no superiority of the tubular technique. Comparative studies did not indicate a significant difference regarding surgical time, bleeding, functional outcomes, and recurrence rates. 21 23 Other studies demonstrate better postoperative pain outcomes, based on the visual analogue scale (VAS) results for low back pain and radiating pain, when comparing conventional and tubular microdiscectomy. 22

Tubular discectomy is therefore a safe and effective alternative for the treatment of lumbar disc herniation. It yields comparable results but has a longer learning curve than conventional microdiscectomy.

### Endoscopic surgery

Endoscopic surgery represents the most significant advance since microdiscectomy. The first descriptions of the technique date from the 1980s and 1990s, but the main advances occurred in the 2000s, with the technological evolution of the instruments. 24 25 The approaches can be performed using a single portal, such as in full-endoscopic discectomy (FED), or biportal technique, as in unilateral biportal endoscopy (UBE), with incisions of 6 to 10mm, and minimal local aggression. 24

Two classic surgical approaches: transforaminal and interlaminar, selected based on the level and morphology of the hernia. 26 The transforaminal route provides direct access to the disc through the Kambin triangle and is the approach of choice for foraminal and extraforaminal hernias, as well as high lumbar levels where the foramen is larger. The interlaminar route favors the treatment of central and central-lateral hernias, especially at low lumbar levels, where the interlaminar window is larger ( Fig. 3 ). 25 26

**Fig. 3:**
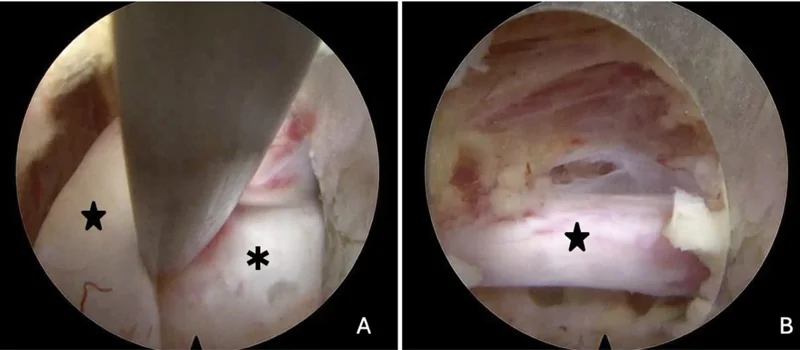
Intraoperative image of lumbar interlaminar endoscopy. ( **A** ) Root retraction and exposure of the disc herniation. ( **B** ) Free nerve after discectomy. ★Lumbar root. ✱Herniated disc.

The clinical results of endoscopic surgery compared with microdiscectomy are similar for postoperative pain (VAS) and functional scores, with the Owestry disability index (ODI) in the medium and long term, with better outcomes for patients undergoing endoscopic surgery. 14 15 16 Other positive factors in favor of endoscopy are a shorter hospital stay, earlier return to work, less intraoperative bleeding, and a lower complication rate. 14 15 27 Regarding recurrence, defined as the return of symptoms at the same level operated on after a symptom-free period, the rates are similar between techniques, ranging from 3 to 5% over a 5-year period. 12 16 27 28

Endoscopic surgery requires specialized instruments and is associated with a higher initial cost than other discectomy options. 29 30 However, early return to work, combined with a shorter hospital stay, means that the total costs of the procedure, including social and productivity costs, tend to be lower for endoscopic surgeries. 29 31 Cost-effectiveness analyses show that endoscopic surgery is more effective and less costly at 12 months than microdiscectomy. 30 31

Endoscopic surgery has a greater learning curve than other techniques. On average, about 25 cases are considered for the biportal technique and 40 cases for the FED, which can increase the complication rate and surgical time in the initial cases. 32 For microdiscectomy, 10 to 18 cases are necessary to achieve proficiency and reduce variability in results. 33

## Regenerative Medicine

The use of regenerative medicine to treat spinal pathologies is still under investigation, with studies focusing on the use of mesenchymal stem cells (MSCs), bone marrow concentrates (BMCs), and platelet-rich plasma (PRP). Several studies seek to evaluate the results of low back pain control in patients with degenerative disc disease, though the results are still insufficient to validate its routine use. 19

For the treatment of patients with radiculopathy due to lumbar disc herniation, there is evidence of better control of radicular pain (VAS) after transforaminal infiltration with PRP compared with corticosteroid use, without changes in function (ODI) or in the control of low back pain. 34

Another regenerative medicine approach evaluated in some studies is the use of PRP after endoscopic discectomy. One of the few randomized clinical trials showed no difference in clinical scores (ODI and VAS) between patients who underwent PRP and those who did not. The only relevant finding of this study was an improvement in the Pfirrmann scale on MRI scans in the group receiving PRP. 35

Despite promising results in other areas, the effectiveness of regenerative medicine in treating lumbar disc herniation remains unclear, with few robust studies and a lack of standardization in application protocols, dosage, and frequency.

## Annular closure devices

Devices and technologies for repairing the annular defect have been developed to address the failure in the FA and prevent recurrence. Among the available systems, annular suture and closure devices stand out. The main indications are for defects above 6 mm with preserved disc height, factors associated with increased herniated disc recurrence. 18

The annular suture can be performed endoscopically or microsurgically. Several suture techniques are described, with bone anchorage, fixation only in the annulus, or joint suture of the posterior longitudinal ligament (PLL). 36 Initial studies demonstrate the safety of the technique, but the results are insufficient to support its routine use in surgical practice. 37

Devices designed to close the annular defect after discectomy are anchoring implants in the vertebral body that fill the annulus gap. Clinical trials demonstrate a reduction in recurrence between 7 and 12% and a drop in reoperation rates from 13 and 16% to 5 and 6%. 18 The rate of serious complications does not increase, and clinical function remains similar between patients with and without the device. 18 Such devices have the Food and Drug Administration (FDA) approval for use in the USA, but are not yet available in the Brazilian market.

## Lumbar arthrodesis

North American guidelines do not recommend arthrodesis for radiculopathy due to disc herniation, being indicated for radiographic instability, multiple relapses, or broad bone decompression. 38 Arthrodesis reduces recurrences and postoperative instability compared with isolated discectomy, especially after multiple recurrences (two or more), but does not improve functional outcomes, as relief is mainly limited to low back pain, with no significant impact on root pain or functional indices. 20 Arthrodesis results in longer surgical time, greater blood loss, prolonged hospitalization, and an increased risk of complications, such as infection, incorrect implant positioning, and degeneration of the adjacent level. 39

Its functional benefit is modest and should be weighed against the higher risk of complications. In manual workers, arthrodesis can relieve low back pain, but results in a lower rate of return to work, longer leave, greater use of analgesic medication, and higher costs, while discectomy offers faster recovery, despite the greater risk of recurrence in physically demanding activities. 37

## Final considerations

The surgical treatment of lumbar disc herniation is constantly evolving, with the development of increasingly advanced techniques. Microdiscectomy remains a safe, effective, widely disseminated, and readily available technique. Endoscopic surgery is increasingly adopted by surgeons and yields superior short-term recovery outcomes, including an early return to function, and is associated with greater cost-effectiveness over 12 months, an important consideration in the current healthcare scenario.

Annular defect closure techniques are still under study, as is regenerative medicine, and quality studies are needed to evaluate their actual effectiveness. Arthrodesis remains an exception in the treatment of herniated discs, indicated only in selected cases.

Finally, the choice of surgical technique should be determined by each surgeon's experience and individualized for each patient, considering the clinical picture, associated risk factors, clinical-radiological correlation, and expected results, in line with the patient's expectations.
